# Blockchain Implementation in Health Care: Protocol for a Systematic Review

**DOI:** 10.2196/10994

**Published:** 2019-02-08

**Authors:** Edward Meinert, Abrar Alturkistani, Kimberley A Foley, Tasnime Osama, Josip Car, Azeem Majeed, Michelle Van Velthoven, Glenn Wells, David Brindley

**Affiliations:** 1 Global Digital Health Unit Department of Primary Care and Public Health Imperial College London London United Kingdom; 2 Healthcare Translation Research Group Department of Paediatrics University of Oxford Oxford United Kingdom; 3 Global Digital Health Unit Department of Primary Care and Public Health, School of Public Health Imperial College London London United Kingdom; 4 Department of Primary Care and Public Health School of Public Health Imperial College London London United Kingdom; 5 Oxford Academic Health Science Centre Oxford United Kingdom

**Keywords:** blockchain, electronic health records, efficiency, interoperability, health, information science, computers

## Abstract

**Background:**

A blockchain is a digitized, decentralized, distributed public ledger that acts as a shared and synchronized database that records cryptocurrency transactions. Despite the shift toward digital platforms enabled by electronic medical records, demonstrating a will to reform the health care sector, health systems face issues including security, interoperability, data fragmentation, timely access to patient data, and silos. The application of health care blockchains could enable data interoperability, enhancement of precision medicine, and reduction in prescription frauds through implementing novel methods in access and patient consent.

**Objective:**

To summarize the evidence on the strategies and frameworks utilized to implement blockchains for patient data in health care to ensure privacy and improve interoperability and scalability. It is anticipated this review will assist in the development of recommendations that will assist key stakeholders in health care blockchain implementation, and we predict that the evidence generated will challenge the health care status quo, moving away from more traditional approaches and facilitating decision making of patients, health care providers, and researchers.

**Methods:**

A systematic search of MEDLINE/PubMed, Embase, Scopus, ProQuest Technology Collection and Engineering Index will be conducted. Two experienced independent reviewers will conduct titles and abstract screening followed by full-text reading to determine study eligibility. Data will then be extracted onto data extraction forms before using the Cochrane Collaboration Risk of Bias Tool to appraise the quality of included randomized studies and the Risk of Bias in nonrandomized studies of Interventions to assess the quality of nonrandomized studies. Data will then be analyzed and synthesized.

**Results:**

Database searches will be initiated in September 2018. We expect to complete the review in January 2019.

**Conclusions:**

This review will summarize the strategies and frameworks used to implement blockchains in health care to increase data privacy, interoperability, and scalability. This review will also help clarify if the strategies and frameworks required for the operationalization of blockchains in health care ensure the privacy of patient data while enabling efficiency, interoperability, and scalability.

**International Registered Report Identifier (IRRID):**

PRR1-10.2196/10994

## Introduction

A blockchain is a digitized, decentralized, distributed public ledger that acts as a shared and synchronized database that records cryptocurrency transactions. While blockchains are essentially decentralized databases, there is no primary ownership of the data [1,2]. Through collaboration, users decide which data are added to the blockchain while ensuring that identical copies of the data are received and automatically updated [2]. Health care, in any setting, generates abundant complex and rich data, ranging from sensitive patient-identifiable data to operational analytics. The dissemination and essential actions of exchanging these health-related data mean that they remain at risk of privacy breaches [3]. Blockchain technologies have been proposed to respond to this challenge [3,4]. Once granted permission, verified users gain access to blockchain systems. This allows them to share relevant data with other verified users, guaranteeing accountability, scalability, and efficiency [3]. While this innovation has shown promise in various sectors, to ensure successful implementation in health care, various challenges need to be addressed first [1,5]. Because health-related data are numerous and sourced from many areas, the integration and linkage of data have the potential to generate valuable population-level insight [6]. A key consideration with the greater integration of health data sources is the need for strategies that safeguard access control to sensitive patient data. Additionally, as there is an expansion of health- and lifestyle-related data resulting from, for example, mobile apps and wearable devices, blockchain technologies may be exploited by patients, providers, and researchers through the enablement of novel mechanisms for consent and access [7].

As blockchains utilize cryptographic techniques to authenticate and verify users, their application may be used to control access to sensitive data [3]. While the adoption of electronic medical records in health care has become the de facto standard, most data within electronic medical records cannot be shared and exchanged between users appropriately [7]. Blockchain technologies, therefore, have the potential to increase interoperability between patients, carers, health care professionals, and researchers through the enablement of novel methods for data linkage of disparate sources [7]. As data can be sourced from one location, blockchains have the potential to tackle storage issues. By recording patient consent, blockchains could be a patient-empowering platform [8,9]. Information flow and exchange between users may only take place once the patient has consented [8]. Consent also allows health care providers to trust the data they access, thereby enabling them to treat their patients accordingly [8].

In addition to ensuring access security, scalability, and data privacy [7], blockchains also have the potential to enhance medical research through various use cases. Via implementation of health record blockchains, data sourced from medical records, health apps, and wearable devices could be stored and made accessible to users throughout their lives [7], thereby facilitating the conduct of longitudinal studies and pharmacovigilance applications. Each time a patient obtains a new prescription or test results, a patient could be notified that new data have been encrypted, sent for storage, and added to an automated system [7]. Moreover, patients would be able to add data sourced from wearable devices and health apps into this system [7]. Once the data are encrypted and stored, researchers can trust the data will not be altered [6]. Patients and participants may consent and revoke access, remaining in control of their information [10]. In addition to facilitating the collection of longitudinal data such as heart rate, diet, and exercise frequency, blockchains may store genomic data [10]. Blockchain technology may also be used to counter prescription drug fraud [10]. For example, Nuco, a blockchain company, addresses prescription duplication and “doctor shopping,” whereby individuals visit numerous physicians to obtain as many prescriptions as they can [10]. According to Nuco, the problem lies in the inadequate communication between physicians and pharmacists, and blockchains have the capacity to tackle this issue through the verification of prescription authenticity [10]. These implementation scenarios show the strengths of implementation of a secure distributed data technology and the benefits they could make for individual and population data analysis.

Before adopting blockchains to empower patients, advance personalized medicine, accelerate research and development, and engage with populations that are considered “hard-to-reach” [7], challenges restricting their implementation need to be addressed. While broader access to health records may be achieved through blockchains, there is limited information on the costs required to establish and operationalize this decentralized framework [11]. Health systems spend large monetary sums on designing and maintaining traditional information system frameworks [11]. Additionally, various resources are required to troubleshoot issues, update parameters, and extract data [11]. Since blockchains do not require frequent troubleshooting, updates, or third-party involvement in financing, it is predicted that implementing blockchain technologies in health care may reduce costs. [11] To ensure adequate performance, organizations and institutions adopting blockchain technologies need to select specific frameworks to establish the size and format of the data that may be added to the system [11]. It may also prove to be a challenge to incentivize those in the health care sector to adopt novel blockchain technologies [11], thus expanding networks and scalability, owing to the unfamiliarity of the distribution authentication technology and concerns regarding ethics and privacy. A potential benefit, however, is that in addition to allowing clinicians access to real-time data, thereby enabling nationwide interoperability and the delivery of more coordinated patient care, researchers will be able to access and monitor nationwide data that could potentially aid in national surveillance and public health. Because using national programs to encourage digital data adoption have been successful [12], it is envisaged that if similar approaches are applied, the uptake of blockchains may also be achieved.

To the best of our knowledge, there are currently no systematic reviews on the strategies utilized to implement blockchains in health care. Nevertheless, a few reviews have been published focusing on specific aspects of blockchains in health care, such as its applications in health care [1], its potential to finance universal health coverage [13], and its potential to tackle counterfeit medicines [14]. The nature of a public distributed ledger also means that while blockchains could be used for a form of authentication and data access, the health care data would not be suitable for storage on a public ledger due to privacy implications. These considerations surround the application and trade-offs in implementation and require further research and potential standards for their use. Despite its potential to improve health care financing, the right systems must be put in place and “appropriate regulatory guidelines” must be followed before blockchains can be used in health care [13]. This is also true for the use of blockchains for tracking medication trade, which was described to be in its “infancy” and in need of further research [14].

Blockchains have the potential to address various challenges pertaining to data in health care. By requiring patient consent and user verification, privacy and security measures are enforced. Interoperability is facilitated, as data are securely shared among those with permission. Storage issues are also addressed through blockchains, as all the data are found in one location. By engaging various users and allowing for the sharing of multiple data sources at once, implementation of this novel approach may allow for more detailed analyses to be conducted, enhancing research and leading to potential disease prevention and health promotion. This aim of this review is to summarize the evidence on the strategies and frameworks utilized to implement blockchains for patient data in health care to ensure privacy and improve interoperability and scalability, with an aim to serve as an evidence base for development of new design innovations.

## Methods

### Systematic Review Execution

The Cochrane protocol guide will be used to guide the development of the systematic review protocol [15]. The Preferred Reporting Items for Systematic Reviews and Meta-Analysis (PRISMA) Protocols (PRISMA-P) 2015 Checklist [16] will be used to report this systematic review (Multimedia Appendix 1). We will define a research question and establish a Population, Intervention, Comparator, and Outcome framework to develop and combine Medical Subject Headings, subject headings, and keywords. The review will undergo the following six stages: literature search, selection of articles, extraction of data, appraisal of quality, analysis of data, and synthesis of data.

### Identification of a Research Question

How do implementation strategies, design, and frameworks required for the operationalization of blockchains in health care enable privacy of patient data while enabling efficiency, interoperability, and scalability?

### Criteria for Considering Studies for the Review

#### Types of Studies

As blockchains applied to the health care sector remain a novel approach, we will not place restrictions on the study type. This review will include all types of studies as long as other eligibility criteria are met, for example, we will consider randomized controlled trials and observational studies. We will only include studies published in English.

#### Types of Participants

The population will consist of patients who have their data incorporated within ecosystems utilizing blockchains in health care data management.

#### Intervention and Comparator

The included studies will assess blockchain technologies in health care systems to improve issues revolving around access, interoperability, and scalability. Dates of publication and study location will not be restricted. Comparators may consist of other traditional frameworks or technological advances adopted in health systems to improve access, interoperability, and scalability, thereby providing more coordinated health care. For example, learning health systems, which utilize data to provide evidence, thereby allowing continuous learning and improvement of health care, may represent a comparator. We will include studies that have not identified a comparator if they meet the remaining criteria required for study inclusion.

#### Types of Outcomes

Textbox 1 outlines the review outcomes. The primary outcomes will include the extent of access, interoperability, scalability of health care blockchains following the implementation of various strategies and frameworks required for their operationalization, impact on computational performance, and costs and benefits for the use of blockchain systems. Health outcomes will be considered as a secondary outcome and will be assessed to determine whether blockchains can improve the health of individuals and populations when compared with more traditional platforms or other technological advances.

We will use Levels of Information Systems Interoperability, a reference model [17], to measure the level of interoperability. The Data Analysis and Synthesis section (below) will discuss details of how to implement this model. We will assess the scalability of the blockchain by measuring blockchain adoption across the study or survey implementation contexts. To assess privacy, we will identify whether the blockchains abide by legal and regulatory frameworks. As regulatory frameworks may vary according to study setting, we will identify relevant frameworks and legislation once studies are selected and the settings identified. As we complete initial scoping of the literature to identify key outcomes relevant for classification in the review, we shall refine the primary and secondary outcomes.

**Review outcomes.**

Primary outcomes:

- Extent of interoperability
- Extent of scalability
- Privacy, security, and access
- Implications and trade-offs of computational performance
- Costs and benefits to be derived from the use of blockchains in existing systems

Secondary outcomes:

- Health outcomes

### Search Methods

We will systematically search the following electronic databases: MEDLINE and PubMed, Embase, Scopus, ProQuest Technology Collection, and Engineering Index (Compendex). Following exploratory research around the review research question, we will develop Medical Subject Headings, subject headings, and keywords. There will be no restrictions placed on dates of publication, study types, and geographic locations. However, we will only include studies published in English. We intend to search MEDLINE and PubMed first by implementing a search strategy for preliminary research (Multimedia Appendix 2). Based on the findings of this search, we will develop our search strategy and will adapt the strategy for the Embase and Scopus databases. We will not restrict the search by date. EndNote X8.2 (Clarivate Analytics, Philadelphia, PA, USA) will be used to import the results of our searches and remove duplicates. The bibliographic citations of included studies will also be manually searched to identify other studies that fill the review’s inclusion criteria. We will also use similar search terms when utilizing search engines such as Google to systematically search the gray literature; we will consider conference proceedings and reports meeting the review criteria.

### Selection of Studies

The titles and abstracts of studies identified following database searches will be screened by two independent reviewers. Upon completion of title and abstract screening, we will assess the remaining studies through full-text reading. Discussion will be used to resolve disagreements. A third reviewer will be consulted if consensus cannnot be reached. The review’s selection process will be demonstrated using a PRISMA flow diagram.

### Data Extraction and Management

Data will be extracted and collated by two independent reviewers onto predetermined data extraction forms. Where reviewers cannot agree following discussion, a third reviewer will be asked to assist in the decision-making process. Data extraction forms will be validated by the review team prior to utilization to ensure acceptability. We will extract the following data:

1. Date of publication and author
2. Characteristics of the study: location, duration, sample size, and control
3. Characteristics of the intervention: departments or facilities adopting the blockchain, blockchain enablers, challenges, costs, and implementation strategies or frameworks
4. Characteristics of the comparator: departments or facilities adopting the comparator, comparator enablers, challenges, costs, and implementation strategies or frameworks
5. Outcomes: extent of access (primary outcome), interoperability (primary outcome), scalability (primary outcome), and health outcomes (secondary outcome).

### Assessment of Risk of Bias of Included Studies

The risk of bias of the included studies will be assessed by two independent reviewers. A third reviewer will assist in the decision making if the two reviewers disagree on their assessments regarding the methodological quality of included studies.

For randomized controlled trials, the Cochrane Collaboration Risk of Bias tool will be used to assess the following [12]: random sequence generation (selection bias), allocation concealment (selection bias), blinding (performance bias and detection bias), incomplete outcome data (attrition bias), selective reporting (reporting bias), and other bias.

Subsequent to the determination of the selection, we will categorize performance, detection, attrition, reporting, and other bias assessments of the included studies as high risk, low risk, or unclear risk. A risk of bias graph and a risk of bias summary will then be developed to illustrate the methodological quality of included studies.

Other nonrandomized studies will be assessed using the Risk of Bias in Non-randomized Studies-of Interventions [18]. This tool will be used to assess the following seven domains [19,20]:

1. Bias due to confounding (preintervention)
2. Bias in the selection of participants to the study (preintervention)
3. Bias in the classification of interventions (at intervention)
4. Bias due to deviations from intended interventions (postintervention)
5. Bias due to missing data (postintervention)
6. Bias in the measurement of outcomes (postintervention)
7. Bias in the selection of the reported result (postintervention)

A qualitative bias framework will be identified during the execution of the review to examine the paper quality of any studies that do not fall under the Cochrane Collaboration Risk of Bias tool or Risk of Bias in Non-Randomized Studies-of Interventions.

### Data Analysis and Synthesis

We intend to summarize our data numerically (by describing the number and type of studies incorporated within the review) and narratively (by synthesizing data from included studies). From the results of our review, we will aim to map the strategies and frameworks enabling operationalization of blockchains within health systems in a clear format. We intend to measure the extent of interoperability using the Levels of Information Systems Interoperability model [17]. Therefore, we will classify the level of interoperability as the following:

1. Enterprise (universal): data are fully shared and distributed across the health system
2. Domain (integrated): data exchange through shared domain-based models
3. Functional (distributed): sharing of logical data models (eg, relational tables) across a health system
4. Connected (peer-to-peer): exchange of data through electronic means
5. Isolated (manual): integration of data from various systems conducted manually.

To assess scalability, we will determine whether studies measured adoption or uptake across the health care sector, thereby enabling us to assess whether nationwide uptake of the automated system is feasible.

In order to assess whether the blockchain addresses privacy and security issues adequately, we will evaluate whether legislation, including the Health Insurance Portability and Accountability Act of 1996 regulations, have been considered [21]. Legislation considered by the review will depend on study settings, and we will identify this upon study selection. If a study uses Health Insurance Portability and Accountability Act of 1996, this will include:

1. Data encryption: whether the system has encrypted information, allowing only those with a “key” to access
2. Audit trail: whether the system stores information on who accessed the information, the application of modifications, and when the system granted access and applied modifications
3. Access control: whether passwords and personal identification numbers are used in the system, limiting access only to those authorized.

## Results

Database searches will be initiated in September 2018. We expect to complete the review in January 2019.

## Discussion

### Principal Findings

By means of the proposed systematic review, we intend to provide evidence of the strategies and frameworks utilized in the implementation of health care blockchains. Through the development of recommendations that will assist key stakeholders in health care blockchain implementation, we predict that the evidence generated will challenge the health care status quo, moving away from more traditional approaches and facilitating decision making of patients, health care providers, and researchers. As the current traditional system applied in health systems does not fully support interoperability, it is predicted that health care blockchains will enable the delivery of team-based health care by means of nationwide interoperability while optimizing precision medicine research and ensuring prescription authenticity. However, prior to large-scale implementation of these automated systems, it is crucial that research and trials ensure that they are cost-effective and secure systems that maintain the privacy and security of patients and comply with regulatory frameworks.

### Strengths and Limitations

A strength of this proposed systematic review is that it will provide evidence on the strategies and frameworks required for the operationalization of efficient health care blockchains. Furthermore, the potential of health care blockchains in enhancing user access, interoperability, scalability, and health outcomes will be assessed. We predict that the unmet needs of patients, health care providers, and researchers regarding data sharing will be identified through conduction of this systematic review. Finally, we predict that areas around the architecture of health care blockchains that require further research will be identified upon completion of the systematic review. A limitation of our review is that studies published in languages other than English will be excluded.

### Conclusions

As systematic reviews provide the highest form of evidence, we anticipate that review findings will provide patients, researchers, and health care providers with information on health care blockchains. Transparent and rigorous methods will be applied, thereby demonstrating replicability of the review. In addition to consulting blockchain experts and professionals, we anticipate that the review will guide the team in developing recommendations pertaining to blockchains that will enable decision making of developers, patients, health care providers, and researchers.

## Appendix

Multimedia Appendix 1 The PRISMA-P 2015 Checklist.

Multimedia Appendix 2 MEDLINE/PubMed search strategy.

## Abbreviations

PRISMA: Preferred Reporting Items for Systematic Reviews and Meta-Analysis
PRISMA-P: Preferred Reporting Items for Systematic Reviews and Meta-Analysis Protocols
