# Supplementary material for: Blockchain Implementation in Health Care: Protocol for a Systematic Review
Source: JMIR Res Protoc. 2019 Feb 8;8(2):e10994. doi: 10.2196/10994 (PMC6384534; doi:10.2196/10994)
Supplement: Multimedia Appendix 2 [file resprot_v8i2e10994_app2.docx]

**APPENDICES**

**Appendix 2:** Table displaying the MEDLINE/Pubmed Search Strategy

| Category | Medical Subject headings (MeSH) | Keywords |
| --- | --- | --- |
| Blockchain technology | _ | blockchain technology  OR blockchain$ OR bitcoin$ OR cryptocurrenc$ OR cryptograph$ OR “ledger technolog$” |
| Healthcare access, interoperability and scalability | Health Information Interoperability,  Health Services Accessibility  Health Care Quality, Access, and Evaluation | Interoper$  OR Access$  OR Quality OR scalab$ OR data privacy |
| Application of included technologies | - | Storage* OR data OR consent OR schema |

## 
